# Supplementary material for: HIVprotI: an integrated web based platform for prediction and design of HIV proteins inhibitors
Source: J Cheminform. 2018 Mar 9;10:12. doi: 10.1186/s13321-018-0266-y (PMC5845081; doi:10.1186/s13321-018-0266-y)
Supplement: Supplementary file 2 — Additional file 2. Source code of HIVProtI web server. [file 13321_2018_266_MOESM2_ESM.zip › HIVprotI_Source-code/hivprotI/weka-3-6-6/doc/weka/gui/explorer/package-summary.html]

weka.gui.explorer


---


|  |  |  |
| --- | --- | --- |
| **Overview** \| **Package** \| Class \| **Tree** \| **Deprecated** \| **Index** \| **Help** \| **Weka's home** | |  |
| **PREV PACKAGE**   **NEXT PACKAGE** | **FRAMES**    **NO FRAMES**     **All Classes** |  |


---

## Package weka.gui.explorer

| **Interface Summary** | |
| --- | --- |
| **Explorer.CapabilitiesFilterChangeListener** | Interface for classes that listen for filter changes. |
| **Explorer.ExplorerPanel** | A common interface for panels to be displayed in the Explorer |
| **Explorer.LogHandler** | A common interface for panels in the explorer that can handle logs |

| **Class Summary** | |
| --- | --- |
| **AssociationsPanel** | This panel allows the user to select, configure, and run a scheme that learns associations. |
| **AttributeSelectionPanel** | This panel allows the user to select and configure an attribute evaluator and a search method, set the attribute of the current dataset to be used as the class, and perform attribute selection using one of two selection modes (select using all the training data or perform a n-fold cross validation---on each trial selecting features using n-1 folds of the data). |
| **ClassifierPanel** | This panel allows the user to select and configure a classifier, set the attribute of the current dataset to be used as the class, and evaluate the classifier using a number of testing modes (test on the training data, train/test on a percentage split, n-fold cross-validation, test on a separate split). |
| **ClustererPanel** | This panel allows the user to select and configure a clusterer, and evaluate the clusterer using a number of testing modes (test on the training data, train/test on a percentage split, test on a separate split). |
| **DataGeneratorPanel** | A panel for generating artificial data via DataGenerators. |
| **Explorer** | The main class for the Weka explorer. |
| **Explorer.CapabilitiesFilterChangeEvent** | This event can be fired in case the capabilities filter got changed |
| **ExplorerDefaults** | This class offers get methods for the default Explorer settings in the props file `weka/gui/explorer/Explorer.props`. |
| **Messages** | Messages. |
| **PreprocessPanel** | This panel controls simple preprocessing of instances. |
| **VisualizePanel** | A slightly extended MatrixPanel for better support in the Explorer. |

---


|  |  |  |
| --- | --- | --- |
| **Overview** \| **Package** \| Class \| **Tree** \| **Deprecated** \| **Index** \| **Help** \| **Weka's home** | |  |
| **PREV PACKAGE**   **NEXT PACKAGE** | **FRAMES**    **NO FRAMES**     **All Classes** |  |


---
